# Fluoxetine Reshapes Macrophage Membrane Sphingolipids and Inflammatory Response Without Affecting Extracellular Vesicle Biogenesis upon Inactivated SARS-CoV-2 Stimulation

**DOI:** 10.3390/membranes16030098

**Published:** 2026-03-04

**Authors:** Jonatan C. S. de Carvalho, Pedro Nobre-Azevedo, Pedro V. da Silva-Neto, Bianca T. M. Oliveira, Lucas A. Tavares, Diana M. Toro, Andrews O. Borges, Murillo A. Nascimento, Eurico Arruda, Ronaldo B. Martins, Fausto Almeida, Carlos A. Sorgi

**Affiliations:** 1 Departamento de Bioquímica e Imunologia, Faculdade de Medicina de Ribeirão Preto—FMRP, Universidade de São Paulo—USP, Ribeirao Preto 14049-900, SP, Brazilpedronobre@usp.br (P.N.-A.);; 2 Departamento de Análises Clínicas, Toxicológicas e Bromatológicas, Faculdade de Ciências Farmacêuticas de Ribeirão Preto—FCFRP, Universidade de São Paulo—USP, Ribeirao Preto 14040-903, SP, Brazil; 3 Centro de Pesquisa em Virologia, Departamento de Biologia Celular e Molecular e Bioagentes Patogênicos, Faculdade de Medicina de Ribeirão Preto—FMRP, Universidade de São Paulo—USP, Ribeirao Preto 14049-900, SP, Brazil; 4 Programa de Pós-Graduação em Imunologia Básica e Aplicada—PPGIBA, Instituto de Ciências Biológicas, Universidade Federal do Amazonas—UFAM, Manaus 69080-900, AM, Brazil; 5 Departamento de Química, Faculdade de Filosofia, Ciências e Letras de Ribeirão Preto—FFCLRP, Universidade de São Paulo—USP, Ribeirao Preto 14040-901, SP, Brazil

**Keywords:** macrophage, sphingolipids, fluoxetine, inflammation, SARS-CoV-2, extracellular vesicles

## Abstract

Sphingolipids (SL) are essential structural and bioactive components of cell membranes, remarkably involved in inflammatory signaling and membrane dynamics. Dysregulation of SL metabolism contributes to pathological inflammation and cellular stress. Selective serotonin reuptake inhibitors (SSRIs), such as fluoxetine (FXT), are known inhibitors of acid sphingomyelinase (aSMase), although their impact on macrophage SL remodeling and inflammatory responses remains unclear. Here, we investigated the modulation of FXT on SL species composition and inflammatory activation in THP-1-derived macrophages stimulated with inactivated SARS-CoV-2 particles, which is a model of viral-induced inflammation. Sphingolipidomic profiling revealed that FXT pre-treatment markedly reduced ceramide (Cer) species while increasing sphingomyelin (SM) and sphingosine-1-phosphate (S1P) levels, consistent with inhibition of the aSMase-Cer axis. These changes were accompanied by attenuation of proinflammatory components, including interleucin (IL)-6, IL-1β, and matrix metalloproteinase (MMP)-9, indicating that SL remodeling correlates with reduced macrophage activation. Despite pronounced alterations in membrane lipid composition, the quantification of extracellular vesicles (EVs) released by FXT-treated macrophages remained unchanged, however the EVs size distribution was smaller compared to non-treated cells. Altogether, our findings demonstrate that FXT reshapes SL metabolism and lipid membrane composition, thereby diminishing macrophage activation without affecting EVs biogenesis. This study emphasizes the immunometabolic role of SL on membrane reprogramming as a mechanism by which pharmacological aSMase inhibition modulates viral inflammation responses.

## 1. Introduction

Eukaryotic cell membranes contain a remarkably diverse repertoire of lipids, and the classical view of membranes as uniform lipid bilayers has evolved, allowing certain lipids to possess self-organizing properties that promote the formation of specialized membrane domains [1]. Sphingolipids (SL) are essential structural components of cell membranes, where they contribute significantly to membrane fluidity, curvature, and the organization of microdomains known as lipid rafts [2]. Lipid rafts are specialized membrane microdomains enriched with lipids, which are characterized as being very heterogeneous and highly dynamic, as well as being responsible for compartmentalizing several cellular processes [3,4,5]. In macrophages and in other cell subtypes, these lipid rafts are principally composed of cholesterol and SL and serve as platforms for receptor clustering, cell signaling, antigen presentation, extracellular vesicles (EVs) biogenesis, and phagocytic functions [6,7]. Disruption of SL homeostasis impairs these functions and can modulate innate immune responses, especially via influencing Toll-like receptor (TLR) signaling and subsequent inflammatory cascades [8].

The SL constitute a complex class of structural lipids of the cell membrane, whose major representatives include ceramides (Cer), sphingomyelins (SM), sphingosines (Sph), and various glycosphingolipids. Indeed, some species of SL are considered bioactive lipids, such as sphingosine-1-phosphate (S1P) and ceramide-1-phosphate (C1P). Despite their structural diversity, SL share interconnected anabolic and catabolic pathways that maintain cellular homeostasis. The SL de novo pathway begins in the endoplasmic reticulum (ER), where serine-palmitoyltransferase catalyzes the condensation of serine and palmitoyl-CoA to produce 3-ketosphinganine. Subsequent reduction and acylation steps yield dihydroceramide, which is desaturated to form Cer, the central hub of SL metabolism. Cer can be further converted into SM, C1P, or glycosphingolipids such as glycosyl- (HexCer) and lactosyl ceramides (LactCer). In parallel, the hydrolysis of SM by acid sphingomyelinases (aSMase) or neutral (nSMase) provides an alternative source of Cer at the cell plasma membrane. Cer can also be degraded to sphingosine (Sph), which may either re-enter the biosynthetic cycle or be phosphorylated by sphingosine kinases to generate S1P, a key lipid involved in immune cell functions. Importantly, distinct Cer species with different acyl chain lengths may differentially contribute to this pathway and exert context-dependent effects on inflammatory, metabolic, and stress-related signaling pathways [9,10,11]. In macrophages, SL metabolism plays a pivotal role in the coordination of inflammatory responses, vesicle release, and membrane remodeling during activation [12].

Acting both as pro-inflammatory and anti-inflammatory mediators depending on the cellular and metabolic context, SL have dual roles in immune response. Cer typically promotes cell death, inflammatory responses, and vascular leak, while S1P generally opposes these actions by supporting cell survival, vascular integrity, and resolution of inflammation [13,14]. In macrophages, inflammatory stimuli, such as lipopolysaccharide (LPS), dynamically remodel the SL network [14]. Moreover, imbalances in the Cer/S1P axis are linked to chronic inflammatory diseases, such as atherosclerosis, fatty liver disease, and neurodegeneration [15,16]. The spatial and temporal regulation of SL species is thus critical for proper inflammatory resolution and prevention of pathological amplification of immune responses [15].

Building upon the concept of membrane microdomains, SL emerged as an important regulator of vesicular trafficking. In particular, Cer generation by SMases promotes negative membrane curvature and domain formation, thereby facilitating membrane invagination and intraluminal vesicle budding within multivesicular bodies (MVB), a fundamental step in EVs biogenesis [17,18]. These EVs comprise a heterogeneous population of membrane-derived structures that include exosomes and larger microvesicles or ectosomes [18]. Although both EV subtypes originate from distinct biogenic pathways, they share similar lipids, proteins, and nucleic acids cargo [18,19,20]. In this context, EVs regulate cell-to-cell communication by transferring lipids, proteins, and nucleic acids, thereby modulating immune signaling, inflammatory responses, tissue homeostasis, and cellular stress adaptation, and contributing to the progression of inflammatory and degenerative disorders [21]. Additionally, the Cer transfer protein CERT is directly involved in regulating both the Cer and SM content of EVs, thus linking SL metabolism to EVs biogenesis through the endosomal sorting complex required for transport (ESCRT) pathway [22,23,24,25,26,27,28]. Changes in SL composition not only modulate the amount of EVs produced but also modify their cargo and functional effects in recipient cells [29]. In addition, SL-enriched EVs from immune cells can influence macrophage polarization and migration, with implications for tissue repair and inflammatory disease progression [30].

Pharmacological inhibition of aSMase has been shown to impair EV formation. Fluoxetine (FXT), a selective serotonin reuptake inhibitor (SSRI) known as a functional inhibitor of aSMase (FIASMA), may therefore alter SL homeostasis and EVs dynamics [31]. This action has several downstream effects, such as diminishing the Cer-rich domains required for viral entry, as observed with SARS-CoV-2 infection, and may modulate inflammatory signaling in both immune and non-immune cells [31]. Furthermore, prolonged FXT treatment leads to SM accumulation and Cer reduction, which is associated with enhanced autophagy and neuroprotection in peri-infarct cortex [32]. Concisely, by targeting SL metabolism, FXT, and other related SSRI have pleiotropic effects that extend beyond neurotransmission, influencing inflammation, cell survival, and potentially, susceptibility of cells to viral infection [31].

In this context, we investigated the effects of FXT on macrophage SL metabolism and EV release stimulated with inactivated virus particles to better elucidate FXT’s broader immunomodulatory mechanisms. Our results show that inactivated SARS-CoV-2 particles induce a robust inflammatory response in macrophages, while FXT treatment attenuates the production of pro-inflammatory components commonly elevated in COVID-19, such as interleukin (IL)-6, IL-1β, and matrix metalloproteinase (MMP)-9, concurrently remodeling the SL composition in plasma and endosomal cellular membranes. Notably, the release and biogenesis of EVs were not significantly affected under FXT treatment conditions. Reshaping SL homeostasis, FXT treatment influences macrophage activation and inflammatory signaling. Overall, we provide new advancements into drug repurposing of FXT in regulating macrophage membrane lipid remodeling, inflammatory signaling, and EV profile, emphasizing its dynamic function beyond being an antidepressant, toward immunometabolic modulation.

## 2. Materials and Methods

### 2.1. Cell Culture

THP-1 monocytes (BRCJ 0234) were maintained and differentiated into macrophages as previously described [33]. For prophylactic exposure, differentiated macrophages were treated with 1 × 10^−7^, 1 × 10^−6^, and 1 × 10^−5^ M of FXT (Cayman, Cat. No 14418-5MG) for 24 h. Following the treatment, cells were washed with an incomplete medium to remove the residual drug. Subsequently, the cells were cultured under these conditions: (I) THP-1 macrophages treated with FXT and stimulated by UV-inactivated SARS-CoV-2 (MOI 2.0) particles (MφV-FXT), (II) THP-1 macrophages not treated with FXT, but stimulated by virus particles (MφV), (III) THP-1 macrophages treated with FXT but not stimulated by virus particles (Mφ0-FXT), and (IV) THP-1 macrophages not treated with FXT and not stimulated by virus particles (double-negative control, Mφ0). The viral inactivation was performed in a biosafety level 3 (BSL-3) facility at the Center for Virology Research—Faculty of Medicine of Ribeirão Preto (FMRP)—*Universidade de São Paulo* (USP), stimulation was carried out for 24 h at 37 °C, 5% CO_2_. Finally, macrophages were washed again and incubated for 24 h in 40 mL of EV-depleted DMEM. For pharmacology control analysis, we included a group treated with an nSMase inhibitor (GW4869—10 μM, Sigma-Aldrich Cat. No. D1692-5MG, Saint Louis, MO, USA), described as an EVs-release inhibitor, to observe EVs release from macrophages in the same experimental conditions as for FXT culture treatment. Culture supernatants from the experimental groups were collected for subsequent isolation and analysis. For comparative inflammatory response experiments, LPS (1 µg/mL, Sigma-Aldrich, Cat. No. L6529-1MG) was used as a positive control. Culture supernatants from the experimental groups were then collected for cytokine and MMP quantification.

### 2.2. Cell Viability Assay

For the cellular cytotoxicity effect, after the treatments carried out on the cell culture plates (96-well—Thermo Scientific Nunc, Suzhou City, China), the cell supernatants were removed and replaced with 100 µL of 3-[4,5-dimethylthiazol-2-yl]-2,5 diphenyltetrazolium bromide solution (MTT—Sigma Aldrich, USA) (0.5 mg/mL), diluted in DMEM-c. After adding this solution, the cells were incubated for 4 h at 37 °C and 5% CO_2_. Then, the culture supernatants were removed, and 100 µL of dimethyl sulfoxide (DMSO—Sigma Aldrich, USA) was added to the wells for 30 min, or until total solubilization of formazan crystals, from the mitochondrial metabolism of MTT. Absorbance measurements were obtained using a microplate spectrophotometer at 570 nm (µQuant—BioTek Instruments, Inc., Winooski, VT, USA). Viability was defined as the ratio (expressed as a percentage) of the absorbance of treated cells relative to untreated cells.

### 2.3. Sphingolipid Extraction

Macrophages (1 × 10^6^ cells) were suspended in 1 mL of phosphate-buffered saline (PBS) and transferred to glass centrifuge tubes. Samples were spiked with 10 µL internal standard (IS—10 µM Cer/Sph Mixture II, LM6005, Avanti Polar Lipids, Alabaster, AL, USA), followed by the addition of 300 µL HCl (18.5%), 1 mL MeOH, and 2 mL CHCl_3_. Samples were vortexed for 30 min (50 rpm) and centrifuged at 2000× *g* for 5 min (modified Folch extraction). After the organic phase was collected, the second extraction was performed on the remaining aqueous phase with another 2 mL of CHCl_3_. Combined organic phases with the extracted lipids were evaporated under vacuum for 45 min at 45 °C. Then resuspended in 50 µL MeOH:CHCl_3_ (4:1, *v*/*v*), vortexed for 1 min, centrifuged for 5 min at 2000× *g*, and the supernatant was injected into the LC-MS/MS system.

### 2.4. LC-MS/MS Quantification of Sphingolipids

Chromatographic separation was performed on an Ascentis Express C18 column (100 × 2.1 mm, 2.7 µm; Supelco, St. Louis, MO, USA) at 40 °C using a Nexera X2 HPLC system (Shimadzu, Kyoto, Japan). Samples (10 µL) were injected after column equilibration for 20 min, as described by our research group [34]. Briefly, a binary gradient system was employed, solvent A (H_2_O with 1% formic acid) and solvent B (MeOH), delivered at 0.5 mL/min with the following program: 0–1 min (30% B), 1.1–2.5 min (85% B), 2.5–5.0 min (100% B), 5.0–15.0 min (100% B), 15.1–20.0 min (re-equilibration at 30% B). Lipids were analyzed using a TripleTOF 5600^+^ mass spectrometer (SCIEX, Redwood City, CA, USA) operated in positive electrospray ionization (ESI) mode with high-resolution multiple reaction monitoring (MRM^HR^). External calibration was performed using an atmospheric pressure chemical ionization (APCI) probe with positive SCIEX calibration solution (SCIEX, Redwood City, CA, USA), maintaining a mass accuracy of <2 ppm. Instrument parameters were as follows: GS1 = 50 psi, GS2 = 50 psi, CUR = 25 psi, ISVF = +4500 V, source temperature = 500 °C, dwell time = 10 ms, mass resolution = 35,000 at *m*/*z* 400. Data acquisition and qualitative analysis were conducted using Analyst^TM^ and PeakView^TM^ version 2.2 softwares (SCIEX, Redwood City, CA, USA). Quantification was performed with MultiQuant^TM^ version 3.0.2 (SCIEX, Redwood City, CA, USA), normalizing peak areas to internal standards. Monoisotopic correction accounted for ^13^C isotope abundance [35]. Final concentrations (pmol/mL) were derived by multiplying the analyte-to-standard peak area ratio by the known standard concentration.

### 2.5. Isolation and Purification of Macrophage-Derived Extracellular Vesicles

All the experiments of isolation and physicochemical analysis of EVs were performed following the Minimal Information for Studies of EV (MISEV2024) guidelines [36]. In brief, cell culture supernatants (40 mL) were clarified by sequential centrifugation at 5000× *g* (15 min) and 15,000× *g* (15 min) at 4 °C (Sorvall Legend XFR, Thermo Fisher, Waltham, MA, USA). The clarified supernatants were concentrated using a 100 kDa Amicon system (Millipore, Burlington, MA, USA) and filtered through 0.45 µm membranes. Filtrates were subjected to ultracentrifugation in an Optima MAX-XP ultracentrifuge using a fixed-angle rotor at 100,000× *g* for 1 h at 4 °C, with acceleration set to 9 and deceleration set to 0 (Beckman Coulter, Brea, CA, USA). The final EV pellet was resuspended in 1 mL ultrapure H_2_O (MilliQ, Darmstadt, Germany) and stored at −80 °C until analysis.

### 2.6. Extracellular Vesicle Characterization

Particle size distribution and concentration were measured by nanoparticle tracking analysis (NTA) using a NanoSight NS300 (Malvern Instruments, Malvern, UK). For each sample, three times 30 s recordings were captured and analyzed to determine mean size and dispersion profiles. For the morphological assessment, EV suspensions were adsorbed onto carbon-coated Transmission Electron Microscopy (TEM) grids (Electron Microscopy Sciences, Hatfield, PA, USA) for 20 min, fixed with 1% glutaraldehyde (Sigma-Aldrich, St. Louis, MO, USA), rinsed with deionized water (MilliQ, Darmstadt, Germany), and visualized using a JEM-2100 microscope (JEOL, Tokyo, Japan), available at Electron Microscopy Multiuser Laboratory at Department of Cell and Molecular Biology (FMRP-USP).

### 2.7. Quantification of Protein Mediators

Secretion of IL-6 (Cat. No. 555157), IL-1β (Cat. No. 557953), MMP-3 (Cat. No. DY513), and MMP-9 (Cat. No. DY911) in culture supernatants was quantified using BD OptEIA™ Human ELISA kits for cytokines (BD Biosciences, Franklin Lakes, NJ, USA) and RD DuoSet-Human ELISA kits for MMPs (R&D System, Minneapolis, MN, USA), Supplementary Table S1, following the manufacturers’ protocol.

### 2.8. Statistical Analysis

Data visualization and statistical comparisons were performed in GraphPad Prism (v9.1, San Diego, CA, USA). Multivariate statistical analyses were conducted using R packages such as *MetaboAnalystR*, *ggplot2*, and *ComplexHeatmap*. Two-way ANOVA followed by Tukey’s post hoc test was applied for parametric comparisons among treatments. One-way ANOVA followed by Tukey’s test was used for comparisons among stimulation conditions. Statistical significance was set at *p* < 0.05, and only significant *p*-values are indicated in figures.

## 3. Results

### 3.1. Viral Stimulation and Fluoxetine Treatment Modulate Macrophage Sphingolipidome

**Figure 1:**
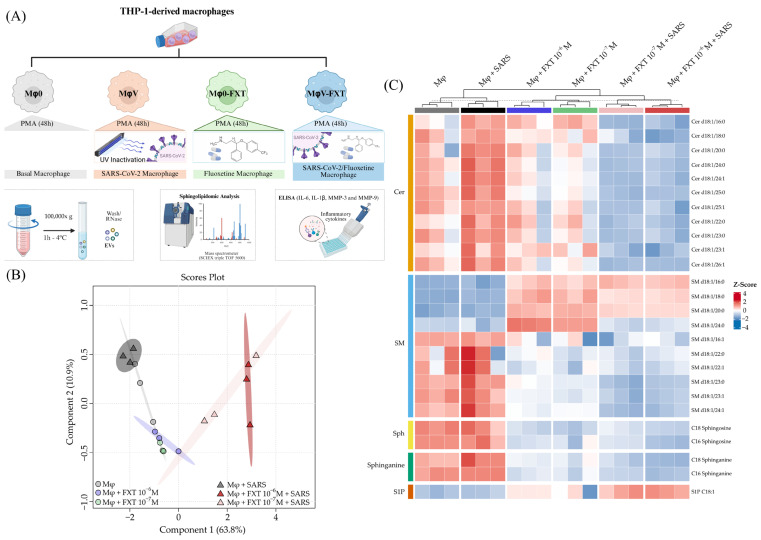
Fluoxetine treatment alters the sphingolipidomic signatures of macrophages under resting and SARS-CoV-2-stimulated conditions. (**A**) Workflow for Mφ differentiation and FXT treatment assay, determination of SL profile, protein mediators quantification, and EV isolation. Created in BioRender (BioRender Inc., Toronto, ON, Canada—Agreement number: QX28ZCZVX3). (**B**) PLS-DA 2D score plot shows the clustering and discrimination among groups based on SL species data (*n* = 3 per group), in which Mφ0 groups are represented as circles, and MφV groups as triangles. The 95% confidence intervals (CI) are indicated as colored areas around samples for each group. (**C**) Heatmap representing relative abundance (*Z*-score) of individual SL species across samples and experimental groups (hierarchical clustering, Euclidean distance).

Figure 1A depicts how FXT treatment affected the sphingolipidome in THP-1-derived macrophages. We observed that both non-stimulated macrophages (Mφ0) and inactivated SARS-CoV-2-stimulated macrophages (MφV) presented a similar overall SL profile, despite the intensity of Cer and SM in the MφV group. However, prophylactic FXT treatment with 1 × 10^−7^ and 1 × 10^−6^ M concentrations (Mφ0 + FXT and MφV + FXT groups) robustly altered cell sphingolipidome, differentially when compared with non-treated groups (Figure 1B). In fact, the FXT concentration used in our experiments corresponded to the highest dose that did not induce cytotoxic effects, as determined by cell viability assays (Supplementary Figure S1). Instead, we analyzed more than 145 SL species across eight different classes by target and quantification approach; the main alterations in the sphingolipidome were observed mostly on Cer, SM, Sph, sphinganines, and S1P metabolites. These lipid species were present at significantly higher levels in the MφV group compared with the other experimental groups (Figure 1C and Supplementary Table S2). Interestingly, S1P levels were completely dependent on FXT treatment, being especially increased in MφV + FXT groups, while Cer, Sph, and sphinganines were strongly reduced in the same groups. We observed a dimorphic pattern in SM abundances, in which SM d18:1/16, SM d18:1/18, SM d18:1/20, and SM d18:1/24 had their levels increased only upon FXT treatment, but the other SM species were completely reduced in the same groups (Mφ0 + FXT and MφV + FXT). Furthermore, the SM d18:1/24:0 species was present only in Mφ0 + FXT. In addition, a two-dimensional Principal Component Analysis (PCA) score plot illustrates the clustering and discrimination among experimental groups based on their SL profile, and the Variable Importance in Projection (VIP) score plot highlights the SL species that most strongly contribute to group discrimination in the multivariate model, as presented in Supplementary Figure S2.

### 3.2. Fluoxetine Shifts Sphingolipid Reprogramming in SARS-CoV-2-Stimulated Macrophages

**Figure 2:**
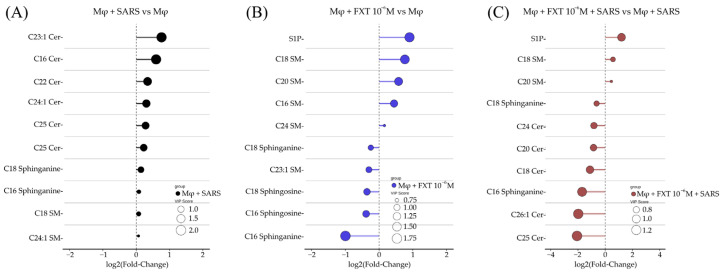
Fluoxetine treatment decreases Cer species and increases S1P production in macrophages stimulated with SARS-CoV-2 particles. Lolliplots showed the top ten SL species, based on *p*-values of the *t*-test, contributing to separation between groups as determined by VIP scores from PLS-DA. (**A**) SARS-CoV-2-stimulated macrophages (MφV) versus non-stimulated controls (Mφ0). (**B**) FXT (10^−6^ M) treatment of non-stimulated macrophages (Mφ0 + FXT, 10^−6^ M) compared to Mφ0. (**C**) SARS-CoV-2-stimulated macrophages with FXT treatment (MφV + FXT 10^−6^ M) compared to MφV. Circle sizes reflect VIP score value, representing the discriminative importance of each lipid species.

We further investigated which specific lipid species were most modulated within each group. To better visualize the magnitude and direction of the sphingolipidomic changes, we provide a representation of statistical comparisons between groups and VIP scores. Each data point represents an individual lipid species, connected to the baseline, allowing a depiction of the degree of alteration relative to the reference condition. This visualization facilitates the identification of the most significantly altered species and emphasizes overall trends in SL remodeling across experimental groups. As shown in Figure 2A, comparison of the MφV and Mφ0 group sphingolipidome revealed that Cer d18:1/23:1 exhibited the greatest upregulation, followed by Cer d18:1/16:0, Cer d18:1/22:0, Cer d18:1/24:1, Cer d18:1/25:0, and Cer d18:1/24:0. These findings indicate a coordinated alteration of multiple Cer species in response to viral stimulation. When comparing the Mφ0 + FXT (10^−6^ M) and Mφ0 groups, FXT treatment increased the levels of S1P, SM d18:1/18:0, SM d18:1/20:0, SM d18:1/16:0, and SM d18:1/24:0, while reducing C18:0 and C16:0 sphinganines, C18:1 and C16:1 Sph, and SM d18:1/23:1 (Figure 2B). In MφV + FXT, (10^−6^ M) compared to MφV (Figure 2C), we observed elevated S1P levels accompanied by increases in SM d18:1/18:0 and d18:1/20:0. However, in contrast to Mφ0, the most pronounced reduction occurred among Cer species, with decreases in Cer d18:1/24:0, d18:1/20:0, d18:1/18:0, d18:1/26:1, and d18:1/25:0 following FXT treatment. These data are consistent with reprogramming of SM-Cer metabolism and SMase-related pathways in MφV and following FXT treatment, as reflected by SM preservation and Cer depletion.

### 3.3. Indirect Pathway Enrichment Analysis Reveals Fluoxetine-Sensitive Sphingomyelinase Activity in Macrophages

**Figure 3:**
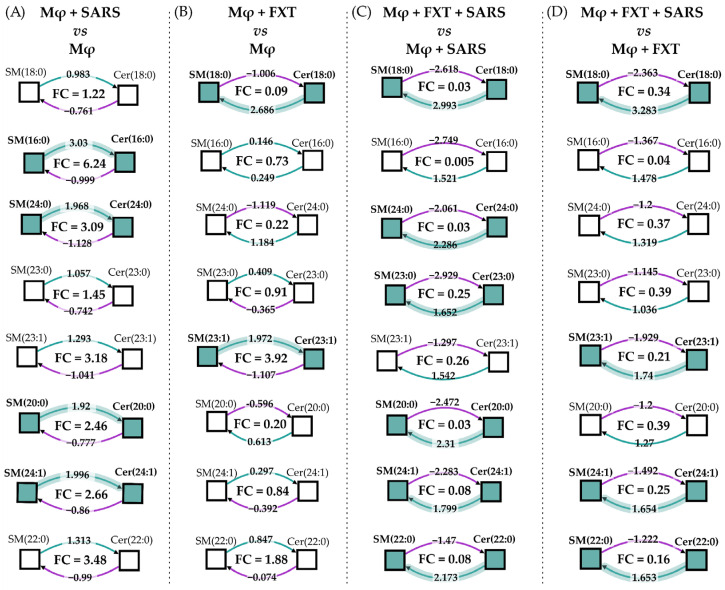
Pathway enrichment analysis of differential sphingomyelinase activity from SM-Cer remodeling in fluoxetine-treated macrophages. Pathway-level enrichment was performed using sphingolipidomic data to indirectly assess SMase activity across experimental conditions by integrating the relative abundance of SM substrates and Cer products within individual reactions of the SM-Cer pathway. For each reaction node, Z-scores were calculated separately for the SM-consuming and Cer-generating directions, and are displayed along the respective arrows, thereby indicating the standardized deviation of each reaction from the reference group in a directional manner. Colored pathways denote statistically significant enrichment (|Z| ≥ 1.645, adjusted *p* < 0.05), whereas non-significant reactions remain uncolored. The central value (FC) at each reaction corresponds to the fold change in the Cer/SM ratio for the indicated SM-Cer pair, comparing (**A**) Mφ + SARS versus Mφ, (**B**) Mφ + FXT versus Mφ, (**C**) Mφ + FXT + SARS versus Mφ + SARS, and (**D**) Mφ + FXT + SARS versus Mφ + FXT. All FXT treatment was made at 1 × 10^−6^ M concentration in macrophage culture. Positive FC values indicate a relative shift toward Cer accumulation (suggestive of increased net SMase-driven flux or reduced SM re-synthesis), whereas negative FC values reflect preservation of SM or depletion of Cer consistent with effective SMase inhibition.

To investigate how FXT modulates the SM/Cer axis, we calculated reaction-level enrichment scores by integrating SM substrates, Cer products, and their corresponding Cer/SM ratios across all experimental contrasts using the BioPAN network tool available on the LIPID MAPS platform (Figure 3). In the comparison between virus-stimulated macrophages (MφV) and control macrophages (Mφ), several long-chain species, particularly those involving SM d18:1/16:0, SM d18:1/20:0, SM d18:1/24:0, and SM d18:1/24:1, exhibited positive Z-scores in the Cer-forming direction, together with Cer/SM fold-change (FC) values greater than 1. This pattern indicates a metabolic shift toward Cer accumulation, consistent with enhanced aSMase activity under viral stimulation (Figure 3A). Prophylactic FXT treatment alone (10^−6^ M; Mφ + FXT vs. Mφ) produced an opposing metabolic profile for many of these reactions, characterized by negative Z-scores toward Cer formation and Cer/SM FC values below 1. This pattern reflects the preservation or accumulation of SM at the expense of Cer generation, even in the absence of viral challenge (Figure 3B).

This SM-preserving profile was further reinforced in virus-stimulated macrophages treated with FXT (MφV + FXT vs. MφV), in which most virus-induced, Cer-biased reactions reverted toward SM enrichment. Consequently, reduced Cer/SM ratios were observed for key species, including (Cer d18:1/23:0)/(SM d18:1/23:0), (Cer d18:1/18:0)/(SM d18:1/18:0), and (Cer d18:1/24:0)/(SM d18:1/24:0) (Figure 3C). The comparison between MφV + FXT and Mφ + FXT (Figure 3D) further demonstrated that FXT modulates virus-associated activation of aSMase-dependent pathways. Relative to the MφV versus Mφ contrast, this comparison revealed a consistent reversal of metabolic flux toward SM preservation in the presence of FXT, indicating effective attenuation of virus-driven Cer production. Notably, only a limited subset of reactions remained oriented toward Cer formation under FXT treatment. For example, the conversion of SM d18:1/23:1 to Cer d18:1/23:1 persisted in the presence of FXT (Figure 3B). This finding suggests that FXT does not completely abolish SMase-related flux, particularly for reactions potentially dependent on nSMase. Instead, FXT preferentially suppresses the most strongly virus-responsive nodes within the pathway, consistent with partial and chain-length-selective inhibition of aSMase activity.

### 3.4. Macrophage-Derived Extracellular Vesicles Production Was Not Modulated by Fluoxetine Treatment

**Figure 4:**
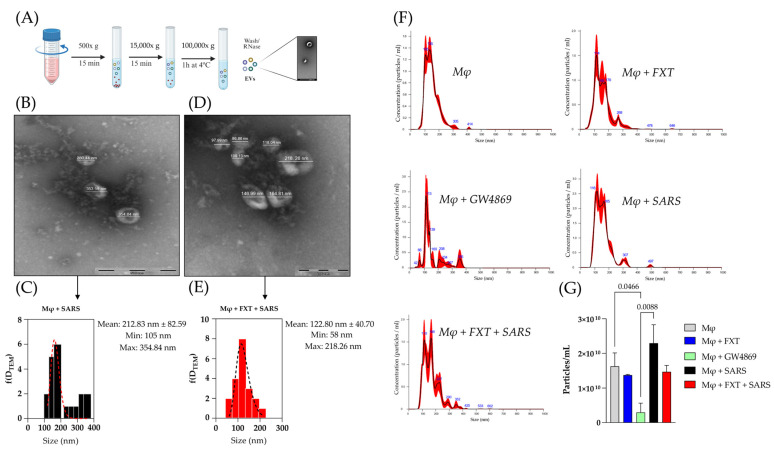
Fluoxetine treatment induces smaller macrophage-derived EVs in size but does not affect biogenesis or release. (**A**) Flowchart for the production and extraction of Mφ-EVs. Created in BioRender (BioRender Inc., Toronto, ON, Canada—Agreement number: KZ28ZD07D9). Representative TEM images and histograms, displaying the size distribution (nm) of EVs isolated from MφV (**B**,**C**) and MφV + FXT 10^−6^ M (**D**,**E**) groups. (**F**) Representative NTA describing the size and concentration profile of isolated Mφ-EVs from the macrophages treated with FXT or GW4869 (1 × 10^−6^ M). (**G**) Concentration of EVs (particles/mL) released in each experimental condition, quantified by NTA (*n* = 3 per group; mean ± SD). Statistical significance was defined at *p* < 0.05, and the value was shown in the graphic bars, calculated by one-way ANOVA with Tukey post-test. Scale bars for TEM are indicated within each image.

Considering the fundamental role of EVs in mediating intercellular communication and propagating inflammatory signals during viral infections, we investigated how treatment with FXT could modulate their biogenesis, morphology, and physicochemical properties (Figure 4A). TEM analyses revealed that FXT treatment significantly reduced the average diameter of EVs released following stimulation with SARS-CoV-2 particles, suggesting an effect of FXT on EV biogenesis or vesicle size distribution (Figure 4B–E).

For NTA, we included a group of EVs from macrophages treated with nSMase inhibitor (GW4869—10 μM), described as an EV-release inhibitor, to be a positive pharmacology control [30]. Although FXT markedly impacted EVs’ size, it did not significantly alter the total concentration of particles produced compared to untreated virus-stimulated macrophages (Figure 4F,G); while treatment with GW4869 significantly reduced EV production compared to the Mφ0 (*p* = 0.0466) and MφV (*p* = 0.0088). Altogether, these results indicate a trend toward altered EV size distribution following FXT treatment, which could potentially impact EV biological function, without affecting total EV release.

### 3.5. Fluoxetine Treatment Suppresses Inflammatory Mediators Released by SARS-CoV-2-Stimulated Macrophages

**Figure 5:**
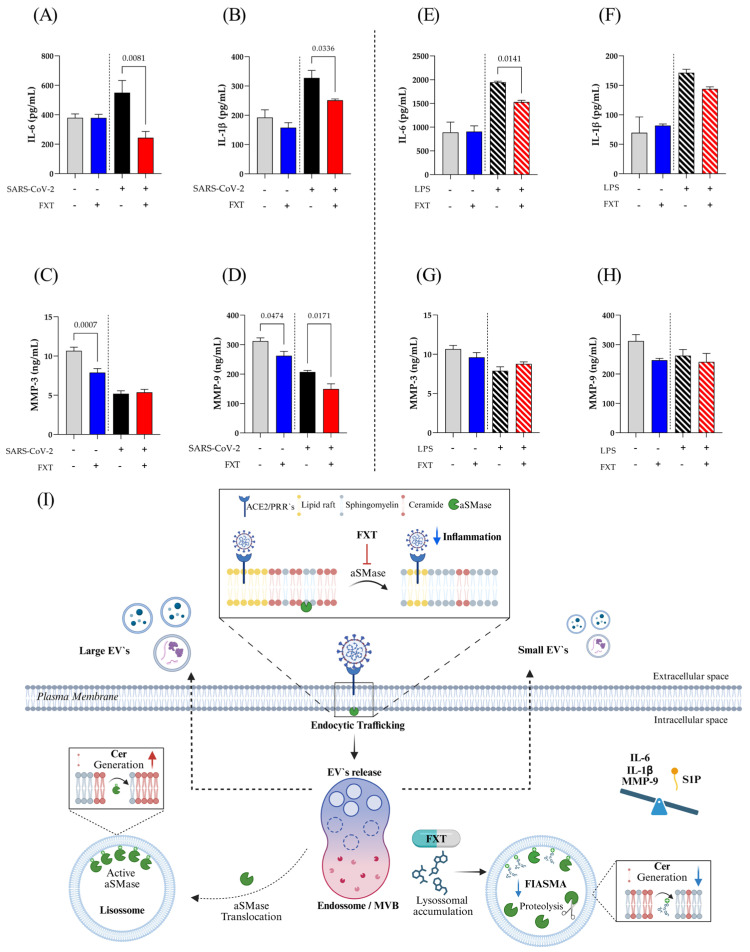
Fluoxetine reduces inflammatory cytokines and MMP-9 secretion by SARS-CoV-2-stimulated macrophages. Protein mediators levels in supernatants from macrophage SARS-CoV-2-stimulated (**A**) IL-6, (**B**) IL-1β, (**C**) MMP-3, and (**D**) MMP-9; or LPS-stimulated (1 μg/mL) (**E**) IL-6, (**F**) IL-1β, (**G**) MMP-3, and (**H**) MMP-9 measured by ELISA (*n* = 4; mean ± SD). Treatment with FXT at 1 × 10^−6^ M (blue bar). Statistical comparisons shown by brackets represent pairwise comparisons between stimulated conditions (SARS-CoV-2 (black bar) or LPS (black dashed bar) in the absence (red bar) or presence (red dashed bar) of FXT. Only statistically significant comparisons (*p* < 0.05) are indicated, as calculated by the *t*-test. Non-significant comparisons were omitted for clarity. (**I**) Schematic representation of the effects of FXT on SL metabolism, inflammatory responses, EVs biogenesis, and release in macrophages under in vitro SARS-CoV-2–stimulated conditions. The diagram highlights the role of aSMase in the hydrolysis of SM to Cer, the S1P formation, the compartmental distribution of these lipids across intracellular and extracellular environments, and the directional trafficking of EVs from the macrophage to the extracellular space. Arrows indicate EV biogenesis and release, as well as lipid remodeling pathways modulated by FXT. Created with BioRender (BioRender Inc., Toronto, ON, Canada—Agreement number: IK29DDL0SP).

To test whether FXT treatment could modulate the production of inflammatory components in SARS-CoV-2-stimulated macrophages, we quantified IL-6, IL-1β, MMP-3, and MMP-9 in cell culture supernatants by ELISA. In this context, Figure 5A,B demonstrated a significant reduction in the release of classic pro-inflammatory cytokines IL-6 and IL-1β, after FXT treatment and SARS-CoV-2-stimulation, important components of the COVID-19 cytokine storm. On the other hand, MMP-3 (Figure 5C) and MMP-9 (Figure 5D) release was significantly decreased by FXT treatment at the macrophage resting level. Notably, all MMP levels were lower in the MφV groups compared to control groups, regardless of FXT treatment. However, we observed a statistically significant reduction in MMP-9 levels of MφV + FXT compared to MφV (*p* = 0.0171). Evaluating other inflammatory stimuli, such as LPS (1 μg/mL), the macrophage treatment with FXT affects only IL-6 levels with a significant reduction (*p* = 0.0141) (Figure 5E). IL-1β levels showed a trend toward reduction in the treated group, although not statistically significant (Figure 5F). Further, MMP-3 (Figure 5G) and MMP-9 (Figure 5H) tend to decrease in cell resting level after FXT treatment, but non-significantly. These results suggest an immunoregulatory effect of prophylactic FXT treatment, attenuating the production of inflammatory mediators induced by different PAMP stimuli, such as SARS-CoV-2 virus particles or LPS.

Our findings indicate that FXT alters the SL composition of the MφV membrane, with reduced Cer levels and increased SM concentrations. Although these changes are directly related to the membrane lipid profile, EV biogenesis was not significantly altered. A shift toward smaller EV particle size was observed, but the functional implications of this change were not directly assessed. In addition, FXT treatment modulated the production of cytokines and MMPs associated with inflammatory activation of macrophage in vitro model. While these data do not elucidate specific regulatory pathways, they provide descriptive insights that may inform future studies, including in vivo investigations, aimed at further exploring the role of FXT in modulating inflammatory responses under viral stimulation (Figure 5I).

## 4. Discussion

Lipid metabolism has emerged as a central regulator of immune responses and EV biogenesis, particularly through the modulation of SL, which influences membrane curvature and the organization of signaling microdomains such as lipid rafts [17,28]. In this study, we demonstrate that prophylactic treatment with FXT, a well-established SSRI and FIASMA, modulates SL-metabolism in macrophages and attenuates inflammatory responses induced by inactivated SARS-CoV-2. By integrating targeted lipidomic and immunochemical analyses, we provide evidence that FXT-induced remodeling of Cer-related pathways reprograms macrophage membrane lipid composition and inflammatory signaling without significantly affecting EV biogenesis. In response to infectious stimuli or cellular injury, aSMase is translocated to the endosomal and plasma membrane, where it catalyzes Cer generation and promotes membrane reorganization [37]. Our results indicate that FXT-mediated modulation of this pathway shifts SL metabolism toward a lipid profile associated with reduced inflammatory signaling [14]. Furthermore, the synthesis and accumulation of Cer at the plasma membrane and endosomal compartments directly influence membrane dynamics, thereby coordinating critical steps in EV biogenesis and cargo sorting [38]. Together, these findings support a model in which FXT selectively alters SL-dependent inflammatory signaling while preserving core mechanisms of EV formation.

The observed increase in SM d18:1/18:0, SM d18:1/20:0 and S1P levels, accompanied by a reduction in Cer d18:1/18:0, Cer d18:1/20:0, Cer d18:1/24:0, is consistent with the potential pharmacological inhibition of aSMase activity, that typical catalyzes the hydrolysis of SM into Cer, a central component in SL metabolism and an important regulator of membrane fluidity, lipid microdomain formation, and signal transduction [39,40,41]. The FXT-mediated shift towards SM accumulation suggests a possible suppression of Cer-mediated inflammatory cascades, since Cer has been shown to facilitate the assembly of receptor complexes involved in NF-κB activation and cytokine release [11,42]. Conversely, S1P is associated with pro-survival and anti-inflammatory signaling pathways mediated by S1P-receptor (S1PR) activation [43]. Our findings support the notion that FIASMA-induced inhibition of aSMase reprograms the Cer–S1P rheostat, thereby promoting a less inflammatory macrophage activation. In addition, this lipid remodeling also influences membrane organization and microdomain architecture, linking SL homeostasis to EVs dynamics and host–pathogen interactions. This connection is particularly evident in lipid raft microdomains, which associate with aSMase in the cell membrane, where their enzymatic activity promotes membrane curvature and vesicle formation [44]. Enveloped viruses, as well as intracellular pathogens, such as *Neisseria gonorrhoeae* [45] and *Trypanosoma cruzi* [46], require aSMase function during cell entry, since these pathogens share invasion mechanisms strongly associated with SM-rich membrane regions [47,48]. SARS-CoV-2, in turn, exhibits an ability to fuse directly with the plasma membrane, thus allowing efficient delivery of its viral genome into the host cell cytoplasm [49].

In this way, several aSMase inhibitors, including antidepressants, have been shown to potently block SARS-CoV-2 entry in vitro [41]. In addition to reducing membrane Cer, these inhibitors induce endolysosomal cholesterol accumulation and disrupt acidification, thus blocking viral entry via the endosomal pathway in a dose-dependent manner [50,51]. In contrast to these effects, sphingosine binds to ACE2 receptors on the membrane, blocking the interaction between ACE2 and the spike protein and consequently inhibiting SARS-CoV-2 entry [52]. Considering that FXT has been shown to suppress the production of pro-inflammatory cytokines, our findings corroborate a broader immunomodulatory role for FXT [53,54]. Our results demonstrated that pretreatment with FXT significantly reduced the secretion of classic pro-inflammatory cytokines, including IL-6 and IL-1β, by SARS-CoV-2-stimulated macrophages in vitro. This anti-inflammatory effect is consistent with previous studies that reported that FIASMA attenuates the cytokine storm and macrophage activation in viral and bacterial infections [55].

Indeed, we extend these observations by linking the reprogramming of macrophage SL composition to the downregulation of inflammatory mediators in response to viral mimetic stimuli. The use of inactivated SARS-CoV-2 provides a relevant model of virus-induced innate activation, allowing the assessment of macrophage lipid remodeling under infectious-like conditions. Studies on septic shock in vivo and allergic asthma have demonstrated that the non-serotonergic anti-inflammatory effects of FXT inhibit NF-κB signaling pathways [56,57], leading to decreased expression of pro-inflammatory cytokines, such as IL-1β, interferon (IFN)-γ, and tumor necrosis factor (TNF), thus exerting a protective effect against chronic inflammation [58]. Furthermore, FXT has been shown to reduce the immunostimulatory properties of tumor cells through macrophage reprogramming in inflammatory environments [59]. Although our study focused on the prophylactic use of FXT, the evidence presented here provides a descriptive purpose that may support future investigations in more complex experimental models.

Although FXT altered the membrane lipid profile, it did not significantly affect the total number of EVs released. Instead, FXT-treated macrophages produced EVs with a reduced size distribution. This observation suggests that, while aSMase inhibition and Cer depletion may modify membrane curvature and biophysical properties, they do not necessarily suppress EV biogenesis. Rather, these alterations appear to influence EVs’ morphology and possibly cargo loading. Given that Cer plays a critical role in exosome formation through endosomal membrane budding, partial inhibition of the aSMase-Cer axis may preferentially affect EVs’ composition and functional properties rather than overall release rates. Accordingly, additional proteomic and lipidomic profiling, together with functional assays in recipient cells, will be required to determine whether FXT-induced EV remodeling translates into altered immunomodulatory activity. Our method for isolating macrophage-derived EVs produced consistent concentrations and morphologies, with particle sizes corresponding to the reference ranges for microvesicles and exosomes [60]. As expected, GW4869 treatment in vitro markedly decreased the number of EVs released by macrophages, which confirms the role of nSMase in reducing the secretion of larger Cer-dependent EVs [61,62]. These findings suggest that variation in EV size, together with SL modulation, may be associated with differences in the inflammatory conditions pathway.

Despite these promising findings, several limitations of this study should be acknowledged. Our experiments were conducted using THP-1–derived macrophages, which, although widely employed as a model for human macrophage responses, may not fully recapitulate the phenotypic heterogeneity and tissue-specific functions of primary macrophage populations in vivo. In addition, the use of inactivated SARS-CoV-2 provides a controlled model of virus-induced inflammation but does not capture the dynamic processes of viral replication and complex host–pathogen interactions that occur during active infection. Furthermore, the functional impact of FXT on EV cargo composition and biological activity remains to be elucidated. Collectively, our findings provide novel evidence that FXT can exert immunomodulatory effects beyond its established role in neurotransmission. By remodeling SL metabolism and membrane architecture, FXT attenuates macrophage inflammatory responses to viral PAMP stimulation without disrupting intercellular communication mediated by EVs. These observations evidence lipid metabolic control points, particularly the aSMase-Cer axis, as promising targets for further investigation in the context of virus-induced inflammatory responses in vivo. Future studies employing more complex experimental models will be necessary to validate and extend these findings. Moreover, comprehensive lipid fluxomics approaches will be essential to elucidate compensatory and auxiliary metabolic pathways that may be engaged during FXT treatment, thereby providing deeper data into the dynamic regulation of lipid networks and their contribution to inflammatory modulation.

## 5. Conclusions

Reprogramming SL metabolism by FXT in macrophages through its activity as a FIASMA leads to the inhibition of the aSMase-Cer axis and consequent accumulation of SM and S1P levels. This lipid remodeling contributes to the re-establishment of SL homeostasis and is associated with a balanced inflammatory state, which was accompanied by reduced production of IL-6, IL-1β, and MMP-9 following stimulation with inactivated SARS-CoV-2 particles, reflecting an attenuated inflammatory macrophage phenotype. Despite extensive alterations in membrane lipid composition, the overall release of EVs remained unchanged, although their size distribution shifted toward smaller vesicles, suggesting potential modifications in cargo. Moreover, this work provides a foundation for future investigations in more complex experimental models aimed at elucidating the translational relevance of SL modulation in viral-associated inflammation.

## Data Availability

The original contributions presented in the study are included in the article. Further inquiries can be directed to the corresponding author.

## Supplementary Materials

The following supporting information can be downloaded at: https://www.mdpi.com/article/10.3390/membranes16030098/s1, Figure S1. Effect of fluoxetine treatment on cell viability in vitro; Table S1. Reagents and chemicals; Table S2. Quantitative sphingolipidomic results; Figure S2. SARS-CoV-2-exposed macrophages display an altered sphingolipid profile compared to non-exposed cells, with higher impact of ceramide levels for clusterization; Figure S3. Pathway enrichment analysis showed differential sphingomyelinase activity from sphingomyelin–ceramide remodeling in 1 × 10^−7^ M fluoxetine-treated macrophages.
