# Supplementary material for: Fluoxetine Reshapes Macrophage Membrane Sphingolipids and Inflammatory Response Without Affecting Extracellular Vesicle Biogenesis upon Inactivated SARS-CoV-2 Stimulation
Source: Membranes (Basel). 2026 Mar 4;16(3):98. doi: 10.3390/membranes16030098 (PMC13027969; doi:10.3390/membranes16030098)
Supplement: Supplementary file 1 [file membranes-16-00098-s001.zip › membranes-4008850-supplementary.pdf]

Supplementary Materials

Fluoxetine Reshapes Macrophage Membrane Sphingolipids and Inflammatory Responses Without Affecting Extracellular Vesicle Biogenesis Upon Inactivated-SARS-CoV-2 Stimulation

Jonatan C. S. de Carvalho [1], Pedro Nobre-Azevedo [1], Pedro V. da Silva-Neto [2], Bianca T. M. Oliveira [1], Lucas A. Tavares [3], Diana M. Toro [3], Andrews O. Borges [4], Murillo A. Nascimento [1], Eurico Arruda [3], Ronaldo B. Martins [2], Fausto Almeida [1], and Carlos A. Sorgi [1,4,5,*]

[1] Departamento de Bioquímica e Imunologia, Faculdade de Medicina de Ribeirão Preto—FMRP, Universidade de São Paulo—USP, Ribeirao Preto 14049-900, SP, Brazil;
[2] Departamento de Análises Clínicas, Toxicológicas e Bromatológicas, Faculdade de Ciências Farmacêuticas de Ribeirão Preto—FCFRP, Universidade de São Paulo—USP, Ribeirao Preto 14040-903, SP, Brazil
[3] Centro de Pesquisa em Virologia, Departamento de Biologia Celular e Molecular e Bioagentes Patogênicos, Faculdade de Medicina de Ribeirão Preto—FMRP, Universidade de São Paulo—USP, Ribeirao Preto 14049-900, SP, Brazil
[4] Programa de Pós-Graduação em Imunologia Básica e Aplicada—PPGIBA, Instituto de Ciências Biológicas, Universidade Federal do Amazonas—UFAM, Manaus 69080-900, AM, Brazil
[5] Departamento de Química, Faculdade de Filosofia, Ciências e Letras de Ribeirão Preto—FFCLRP, Universidade de São Paulo—USP, Ribeirao Preto 14040-901, SP, Brazil
* Correspondence: carlos.sorgi@usp.br

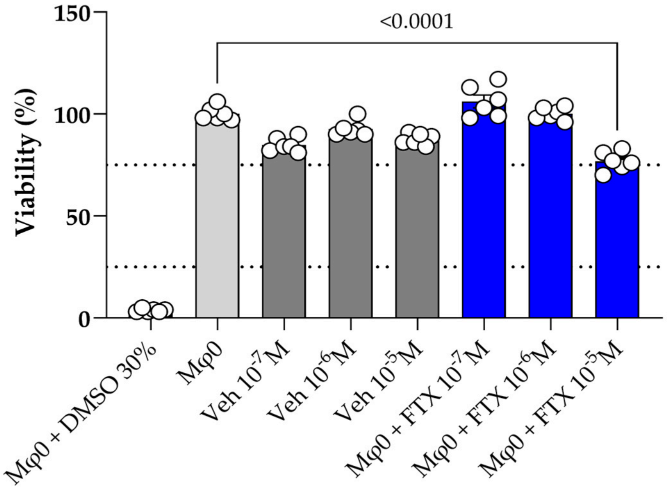

Figure S1. Effect of fluoxetine treatment on cell viability in vitro. Cell prediction assay under different FXT concentrations (n=6). Statistical analyses were performed with a one-way ANOVA test, followed by Tukey's post-test, considering significance at $p < 0.05$.

Table S1. Reagents and chemicals**.**

Products	Brand	Catalog No:
Human IL-6 ELISA Set II	BD OptEIA™	555220
Human IL-1β ELISA Set II	BD OptEIA™	557953
Human Total MMP-3 DuoSet ELISA	R&D Systems	DY513
Human MMP-9 DuoSet ELISA	R&D Systems	DY911
LPS (lipopolysaccharide)	Sigma-Aldrich	L6529-1MG.
Fluoxetine	Cayman	14418-5MG
GW4869	Sigma-Aldrich	D1692-5MG
Forbol 12-miristato-13-acetato	Sigma-Aldrich	P1585-1MG

Table S2. Quantitative sphingolipidomic results.

SL species	Mφ	Mφ + SARS	Mφ + FXT (10^{-7}M)	Mφ + FXT (10^{-6}M)	Mφ + FXT (10^{-7}M) + SARS	Mφ + FXT (10^{-6}M) + SARS
Cer d18:1/16:0	61.19 ± 32.9	390.78 ± 99.88*	254.52 ± 82.81	174.58 ± 93.96	7.19 ± 0.61#&	8.41 ± 3.65#
Cer d18:1/18:0	43.82 ± 23.1	60.84 ± 5.16	32.27 ± 11.5	28.35 ± 1.74	13.57 ± 5.36#	6.7 ± 0.91#
Cer d18:1/20:0	55.56 ± 37.6	158.72 ± 16.03*	45.42 ± 13.55	58.99 ± 32.5	13.75 ± 0.42#	16.67 ± 3.17#
Cer d18:1/24:0	1062.8 ± 444.75	3288.3 ± 1203.9*	325.96 ± 98.55	423.39 ± 228.15	87.16 ± 5.83#	93.51 ± 16.6#
Cer d18:1/24:1	680.70 ± 395.05	2436.6 ± 601.44*	251.92 ± 43.7	382.2 ± 184.63	56.55 ± 2.22#	65.23 ± 18.56#
Cer d18:1/25:0	133.14 ± 56.15	377.74 ± 168.5*	25.94 ± 9.25	22.12 ± 9.76	3.64 ± 0.69#	4 ± 0.05#
Cer d18:1/25:1	21.36 ± 10.56	30.61 ± 2.4	7.12 ± 4.19	9.42 ± 6.56	1.17 ± 0.03#	1.31 ± 0.67#
Cer d18:1/22:0	89.449 ± 21.5	307.48 ± 113.6	115.13 ± 61.14	141.29 ± 93.2	22.85 ± 3.56#	21.03 ± 4.36#
Cer d18:1/23:0	152.47 ± 95.34	292.77 ± 54.78	64.95 ± 29.32	81.77 ± 48.48	16.23 ± 1.01#	18.25 ± 5.94#
Cer d18:1/23:1	5.04 ± 1.29	17.9 ± 9.87	8.53 ± 4.46	6.84 ± 1.84	1.08 ± 0.38#	0.85 ± 0.33#
Cer d18:1/26:1	33.74 ± 14.85	79.24 ± 32.04	13.13 ± 3.67	18.02 ± 9.87	3 ± 0.26#	2.96 ± 0.07#
SM d18:1/16:0	53.54 ± 1.32	53.13 ± 8.09	221.48 ± 22.64*	228.08 ± 34.5*	254.38 ± 23.99#	245.46 ± 16.06#
SM d18:1/18:0	16.4 ± 0.31	18.98 ± 0.87	112.89 ± 21.91*	118.28 ± 22.72*	73.63 ± 3.13#	79.03 ± 5.12#
SM d18:1/20:0	24.1 ± 2.71	25.89 ± 1.57	120.5 ± 3.16*	115.06 ± 6.85*	87.95 ± 2.77#&	86.14 ± 0.5#§
SM d18:1/24:0	129.36 ± 1.34	126.84 ± 12.26	208.51 ± 0.04*	228.04 ± 4.94*	133.73 ± 5.94&	137.76 ± 3.84§
SM d18:1/16:1	227.62 ± 10.14	243.91 ± 43.19	65.43 ± 46.17*	68.84 ± 40.97*	36.8 ± 20.78#	49.67 ± 19.29#
SM d18:1/22:0	190.84 ± 40.25	212.4 ± 80.35	138.11 ± 7.09	153.95 ± 5.91	134.12 ± 9.33	143.95 ± 4.76
SM d18:1/22:1	125.72 ± 31.24	148.34 ± 54.2	74.24 ± 13.89	76.85 ± 7.42	71.99 ± 7.29	72.46 ± 5.22
SM d18:1/23:0	138.98 ± 7.32	190.25 ± 47.71	68.42 ± 0.2*	79.74 ± 5.64*	41.65 ± 4.48#	47.812 ± 1.24#
SM d18:1/23:1	316.26 ± 48.48	368.97 ± 138.24	90.99 ± 7.95*	105.63 ± 2*	54.62 ± 11.92#	59.46 ± 4.82#
SM d18:1/24:1	440.04 ± 21.13	605.05 ± 136.17	298.93 ± 1.28	288.35 ± 14.43	191.19 ± 9.12#	204.2 ± 15.07#
Sph d18:1	841.49 ± 74.05	790.99 ± 253.29	164.72 ± 19.25*	191.47 ± 19.64*	228.07 ± 22.77#	173.82 ± 5.83#
SM d16:1	77.77 ± 0.74	77.3 ± 15.53	29.85 ± 8.4*	28.16 ± 1.18*	29 ± 0.73#	20.65 ± 0.85#
Sphinganine d18:0	107.7 ± 7.03	178.05 ± 37.02	52.69 ± 10.43*	51.85 ± 2.11*	38.03 ± 5.58#	27.87 ± 0.7#
Sphinganine d16:0	1555.03 ± 401.21	2269.3 ± 129.14	50.23 ± 27.85*	40.51 ± 10.97*	22.29 ± 1.92#	10.97 ± 1.79#
S1P C18:1	9.17 ± 1.71	10.93 ± 1.99	41 ± 31.12	60.64 ± 0.23	199.46 ± 81.13&	236.913 ± 48.13§

*****, $p < 0.05$ vs M0. #, $p < 0.05$ vs MV. **&**, $p < 0.05$ vs M0 + FXT 10-7M. §, $p < 0.05$ vs M0 + FXT 10-6M. Data is presented in pmol/mL as mean ± standard deviation.

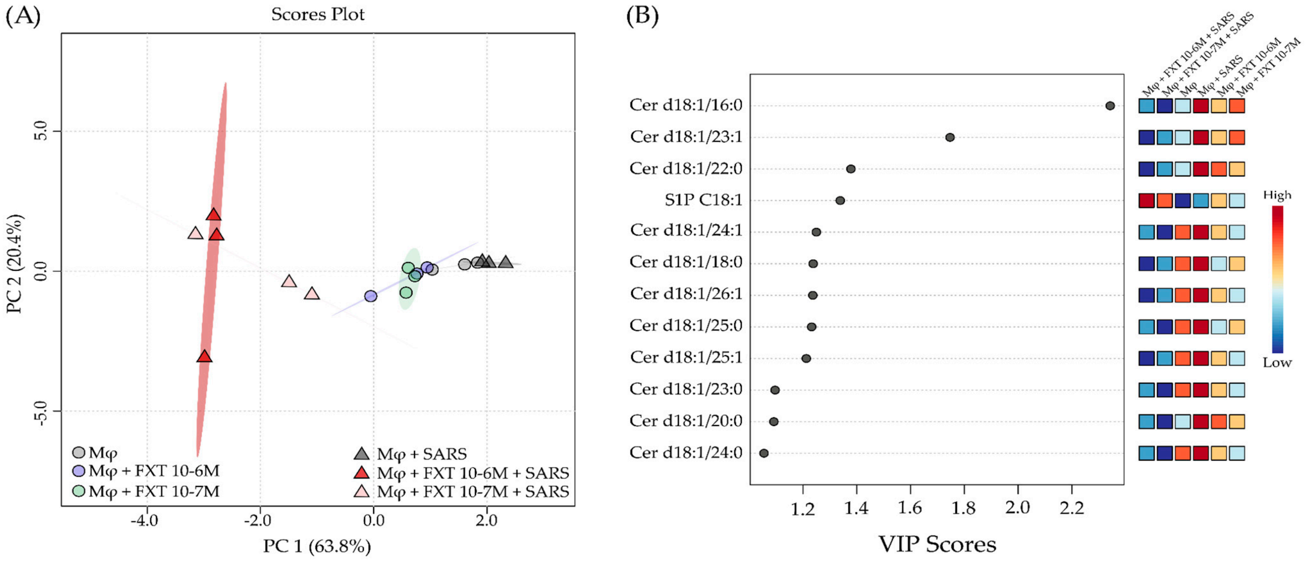

Figure S2. SARS-CoV-2-exposed macrophages display an altered sphingolipid profile compared to non-exposed cells, with a higher impact of ceramide levels for clusterization. (A) Principal Component Analysis (PCA) 2D score plot shows the clustering and discrimination among groups based on SL species data (*n* = 3 per group), in which Mφ groups are represented as circles, and Mφ + SARS-CoV-2 groups as triangles. The 95% confidence intervals (CI) are indicated as colored areas around samples for each group. (B) Variable Importance in Projection (VIP) scores plot for the 12 most important SL species for group discrimination in PLS-DA analysis.

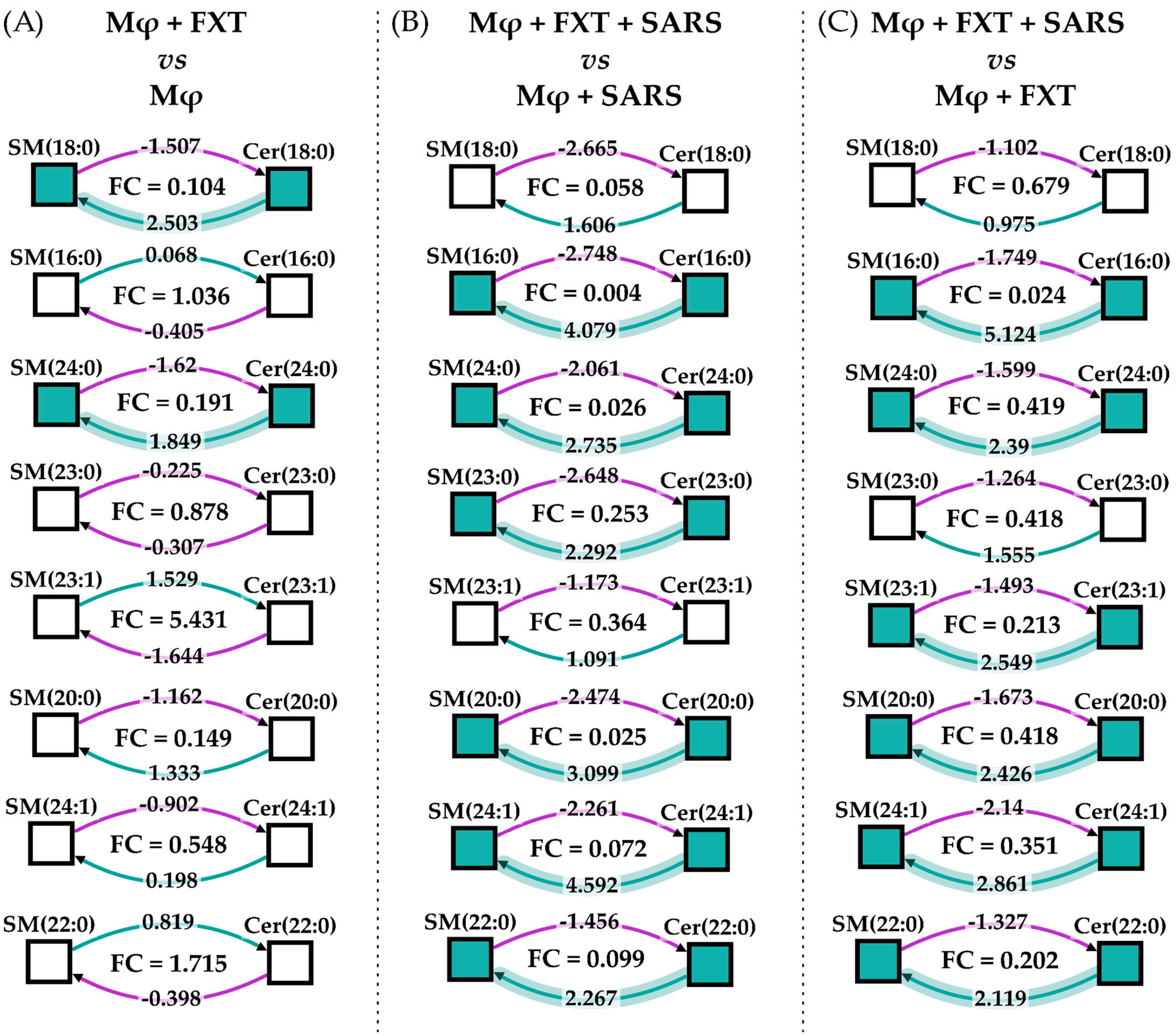

Figure S3. Pathway enrichment analysis showed differential sphingomyelinase activity from sphingomyelin–ceramide remodeling in 10^{-7}M fluoxetine-treated macrophages. Pathway-level enrichment was performed using sphingolipidomic data to indirectly assess SMase activity across experimental conditions by integrating the relative abundance of SM substrates and Cer products within individual reactions of the SM–Cer pathway. For each reaction node, Z-scores were calculated separately for the SM-consuming and Cer-generating directions, and are displayed along the respective arrows, thereby indicating the standardized deviation of each reaction from the reference group in a directional manner. Colored pathways denote statistically significant enrichment ($|Z| \geq 1.645$, adjusted $p < 0.05$), whereas non-significant reactions remain uncolored. The central value (FC) at each reaction corresponds to the fold change in the Cer/SM ratio for the indicated SM–Cer pair, comparing **(A)** Mφ + FXT versus Mφ, **(B)** MφV + FXT versus MφV, and **(C)** MφV + FXT versus Mφ + FXT. Positive FC values indicate a relative shift toward Cer accumulation (suggestive of increased net SMase-driven flux or reduced SM re-synthesis), whereas negative FC values reflect preservation of SM or depletion of Cer consistent with effective SMase inhibition.
